# A xyloglucan endotransglucosylase/hydrolase gene, *IbXTH16*, increases cold tolerance in transgenic sweetpotato

**DOI:** 10.3389/fgene.2025.1629260

**Published:** 2025-06-18

**Authors:** Tao Yu, Jiaquan Pan, Sitong Liu, Zitong Yang, Zhenlei Liu

**Affiliations:** Crop Research Institute, Liaoning Academy of Agricultural Sciences, Shenyang, China

**Keywords:** sweetpotato, cold tolerance, *IbXTH16*, BR, proline

## Abstract

**Introduction:**

Low temperature is a key environmental factor that threaten sweetpotato growth and development. In-depth studies on the gene functions underlying cold resistance are important for genetic engineering in sweetpotato.

**Methods:**

The *IbXTH16* gene was cloned using a homologous cloning approach. Its expression was detected in sweetpotato leaves subjected to low-temperature stress and brassinosteroid treatment. Subsequently, the *IbXTH16* gene was introduced into sweetpotato variety Lizixiang to generate *IbXTH16*-overexpressing plants, thereby enabling the functional validation of the *IbXTH16*.

**Results and discussion:**

The *IbXTH16* gene was cloned from the cold-tolerant variety LHS21. Its 879 bp coding sequence encoded a 292 aa protein with a molecular weight of 32.983 kDa and a pI of 8.47. The 2039 bp genomic sequence of *IbXTH16* contained two exons and one intron. The IbXTH16 protein was localized in the cell membrane. *IbXTH16* was strongly induced by 4°C and brassinosteroid. *IbXTH16* positively regulates cold tolerance of sweetpotato by activating the BR and proline pathways.

## 1 Introduction

Low temperature is a key environmental factor that threaten crop growth and development worldwide (Peng et al., 2014; Shi et al., 2018). Sweetpotato, *Ipomoea batatas* (L.) Lam., an important cash crop, serves as both a staple food and a bioenergy resource (Zhang et al., 2019). As sweetpotato is native to tropical America, it exhibits sensitivity to low temperature, highlighting the importance of enhancing cold tolerance to ensure sustained productivity (Jin et al., 2017; Yu et al., 2022). The development and cultivation of low-temperature tolerant sweetpotato varieties hold significant importance for addressing temperature-related challenges and ensuring global food security. Therefore, in-depth studies on the gene functions underlying cold resistance are important for genetic engineering in sweetpotato.

The xyloglucan endotransglycosylase/hydrolase (XTH) superfamily is an important protein widely present in plants, mainly catalyzing the endohydrolysis of the β-1,4 glycosidic bond of xyloglucan and the self-connection of the xyloglucan molecule (Rose et al., 2002; Morales-Quintana et al., 2020). The XTH superfamily has been reported to participate in diverse biological processes of plants such as fruit maturation and drought response (Miedes et al., 2010; Wu et al., 2022; Han et al., 2023). In cold stress, *AtXTH21* positively modulated the freezing stress resistance and *XTH19* mutant exhibited reduced freezing tolerance in *Arabidopsis* (Shi et al., 2014; Takahashi et al., 2021). In sweetpotato, under cold treatment, only *IbXTH02* and *IbXTH12* of the XTH family were downregulated expression (Zhang et al., 2023). However, XTHs’ function on cold stress of sweetpotato remain largely unknow.

Brassinolide (BR) refers to a group of polyhydroxy steroids that include BR and its structural analogs (Kim and Russinova, 2020). As a type of steroid hormone, BR is ubiquitously distributed across various plant tissues (Xu et al., 2020). Previous studies have demonstrated that the exogenous application of BR can enhances plant cold tolerance. Specifically, treatment with 2 mg L^-1^ BR significantly mitigated leaf surface damage in rice plants, improving their resistance to cold stress (Wang et al., 2020). Under exogenous BR treatment, mangoes exhibited an increased proportion of unsaturated fatty acids in cell membranes, which enhanced membrane fluidity and consequently improved cold tolerance (Li et al., 2012). In addition to exogenous BR treatment, endogenous BR signalling pathways in plants also play a crucial role in regulating cold tolerance. The homologous protein CES of brassinosteroid enhanced expression, which acts as a positive regulatory factor in BR signal transduction, can directly interact with downstream CBF proteins and activate the transcription of *CBF1* and *CBF3*, contributing to enhancing plant cold tolerance (Eremina et al., 2016). Overexpression of the BR receptor *BRI1*, a key activator of BR signalling, has been shown to improve the cold tolerance of tomato plants (Wang et al., 2022). Conversely, BIN2, a negative regulator of BR signalling in *Arabidopsis*, negatively regulates plant cold tolerance by modulating the activities of BZR1 and phosphorylated ICE1 (Ye et al., 2019).

Proline serves as a critical osmotic regulatory compound that is ubiquitously present in plants and plays a protective role under low temperature stress conditions (Kidokoro et al., 2022; Kim et al., 2024). Specifically, proline helps maintain the stability of biological membranes and various enzymes while regulating the acid-base and redox balance within the cytoplasm (Takagi, 2008; Hayat et al., 2012). In *Arabidopsis*, the content of proline increases with prolonged exposure to 4°C low temperature treatment (Kaplan et al., 2007). In cucumber, the *ICE1* enhances the cold tolerance of transgenic plants by promoting the accumulation of free proline (Liu et al., 2010). In *Rosa multiflora*, RmZAT10 specifically binds to and activates the promoter of *RmP5CS*, thereby regulating proline biosynthesis and positively influencing cold resistance (Luo et al., 2022).

In this study, a XTH superfamily gene *IbXTH16* was cloned and characterized from sweetpotato. The *IbXTH16* gene was introduced into the sweetpotato variety Lizixiang to verify its function. Functional analysis showed that overexpression of *IbXTH16* enhanced cold tolerance of sweetpotato by activating the BR and proline pathways.

## 2 Materials and methods

### 2.1 Plant materials

The sweetpotato cold-tolerant variety Liaohanshu21 (LHS21) was employed to clone the *IbXTH16* gene. The sweetpotato variety LHS21 and cold-susceptible variety Sushu28 (SS28) were employed to analyze the expression level of *IbXTH16*. The sweetpotato variety Lizixiang was used to identify the function of *IbXTH16*.

### 2.2 Cloning and sequence analysis

Total RNA for cDNA generation (TRIzol reagent, CWBIO, Beijing, China) and genomic DNA (Easy Pure Plant Genomic DNA Kit, Trans Gen, Beijing, China) were isolated from the leaves of LHS21 according to Fan et al. (2024). The coding sequence (CDS), genome sequence, and promoter region of *IbXTH16* were obtained based on a homologous cloning approach. Phylogenetic analysis was performed with MEGA 11.0 software. The genomic structure of *IbXTH16* was analyzed by GSDS 2.0 (http://gsds.gao-lab.org/). The cis-acting regulatory elements of the *IbXTH16* promoter region were analyzed by PlantCARE (https://bioinformatics.psb.ugent.be/webtools/plantcare/html/). All primers in this study were showed in Supplementary Table S1.

### 2.3 Subcellular localization

The CDS of *IbXTH16* (without the stop codon) was integrated into the pCAMBIA1300-*GFP* vector according to Zhang et al. (2020). The pCAMBIA1300*-IbXTH16*-*GFP* was introduced into *Agrobacterium tumefaciens* strain GV3101 and transiently inoculated into *Nicotiana benthamiana* leaf hypodermal cells. After 48 h of infection, the GFP signals were observed using a confocal fluorescence microscope (LSM880, Zeiss, Jena, Germany).

### 2.4 Expression analysis

The four-week-old *in vitro*-grown LHS21 or SS28 plants was treated with cold (4°C) or 100 mM BR for 0, 1, 3, 6, 12, 24 h and the expression of *IbXTH16* was quantified with Real-time quantitative polymerase chain reaction (RT-qPCR) (SYBR Green Master Mix, YEASEN, Shanghai, China). Expression of *IbXTH16* in leaf, root, and stem tissues four-week-old *in vitro*-grown LHS21 was quantified with RT-qPCR. The *IbACTIN* was used as the internal control.

### 2.5 Production of transgenic sweetpotato plants

The CDS of *IbXTH16* (without the stop codon) was integrated into the pCAMBIA1300 vector according to Fan et al. (2024). The pCAMBIA1300*-IbXTH16* was introduced into *A*. *tumefaciens* strain EHA105, and then infected Lizixiang embryogenic suspension cultures as described by Yu et al. (2007). The transgenic plants were identified with PCR (LA Taq, TaKaRa, Tokyo, Japan) and RT-qPCR.

### 2.6 Cold tolerance analysis

4-week-old *IbXTH16*-overexpressing sweetpotato plants and wide type (WT) with the same status were subjected to cold treatment after a week of acclimatization. The cold treated leaves of the *IbXTH16*-overexpressing sweetpotato plants and WT were used to determine the superoxide dismutase (SOD) (SOD-1-W, Cominbio, Suzhou, China) and peroxidase (POD) activities (POD-1-Y, Cominbio), proline (PRO-1-Y, Cominbio) and malondialdehyde (MDA) contents (MDA-1-Y, Cominbio) according to manufacturer’s instructions. The BR content and relative electrical conductivity were determined by Norminkoda Biotechnology Co., Ltd (Wuhan, China). The expression of *IbDWF4*, *IbDET2*, *IbBRI1*, *IbBES1*, *IbBEE3*, *IbBIN2*, *IbP5CR*, *IbP5CS*, *IbP5CDH*, and *IbPDH* were quantified with RT-qPCR.

### 2.7 Statistical analysis

Data are analyzed using one-way ANOVA followed by *post-hoc* Tukey’s test or Student’s *t*-test at *P* < 0.05 or *P* < 0.01.

## 3 Results

### 3.1 Cloning and sequence analysis of *IbXTH16* and its promoter

**FIGURE 1:**
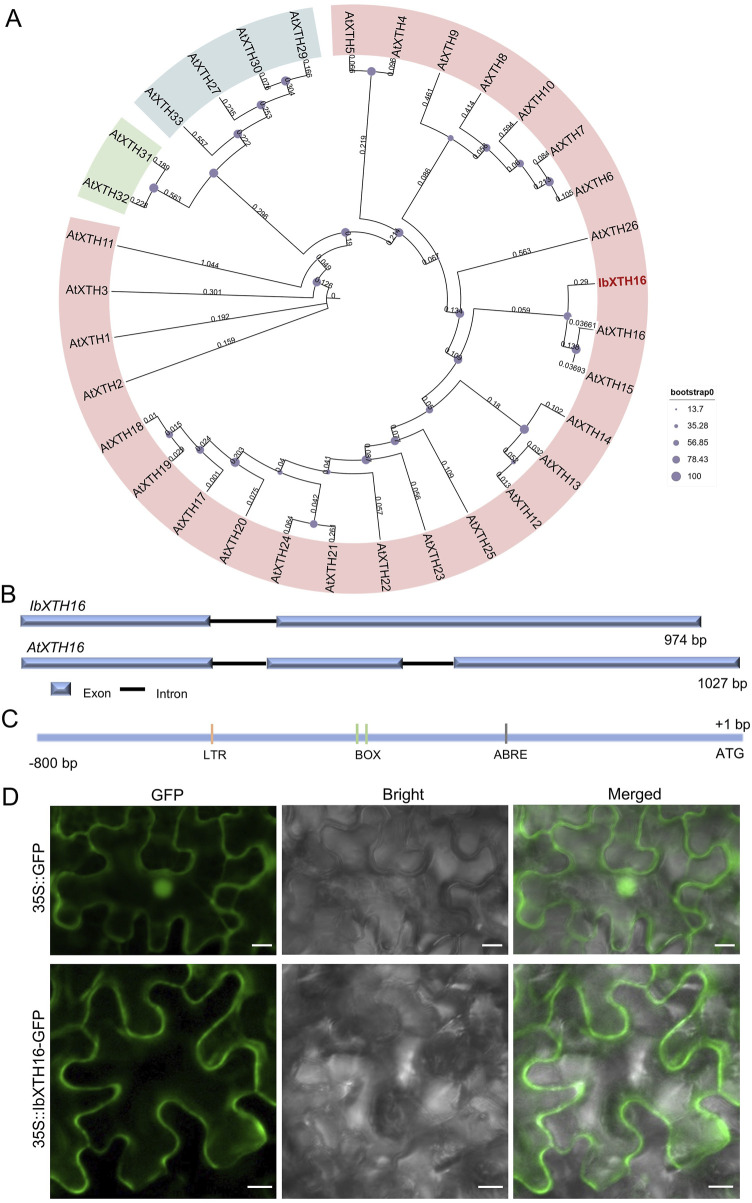
Sequence analysis and subcellular localization of IbXTH16. **(A)** Phylogenetic tree of IbXTH16 and XTH family of *Arabidopsis*. The IbXTH16 cloned in this study is marked in red. **(B)** Comparison of *IbXTH16* and *AtXTH16* genomic structures. **(C)** Diagrammatic representation of the *IbXTH16* promoter. **(D)** Subcellular localization of IbXTH16 in *N. benthamiana* leaf hypodermal cells. Bars = 10 μm.

To identify potential regulators of cold resistance in sweetpotato, we cloned *IbXTH16* gene from cold-tolerant variety LHS21. Its 879 bp CDS encoded a 292 aa protein with a molecular weight of 32.983 kDa and a *p*I of 8.47 (Supplementary Table S2). Phylogenetic analysis showed that IbXTH16 shared the closest relationship with AtXTH16 among *Arabidopsis* homologs (Figure 1A). The 2039 bp genomic sequence of *IbXTH16* contained two exons and one intron, which was different from the three exons and two introns of *AtXTH16* (Figure 1B). The 800 bp *IbXTH16* promoter region contained a low-temperature response element LTR and an ABA-response element ABRE (Figure 1C). To examine the subcellular location of IbXTH16, the IbXTH16-GFP fusion protein was conducted by transiently expressing in *N. benthamiana* leaf epidermal cells. The results showed that IbXTH16-GFP was localized in the cell membrane (Figure 1D).

### 3.2 Expression analyses of *IbXTH16* in sweetpotato

**FIGURE 2:**
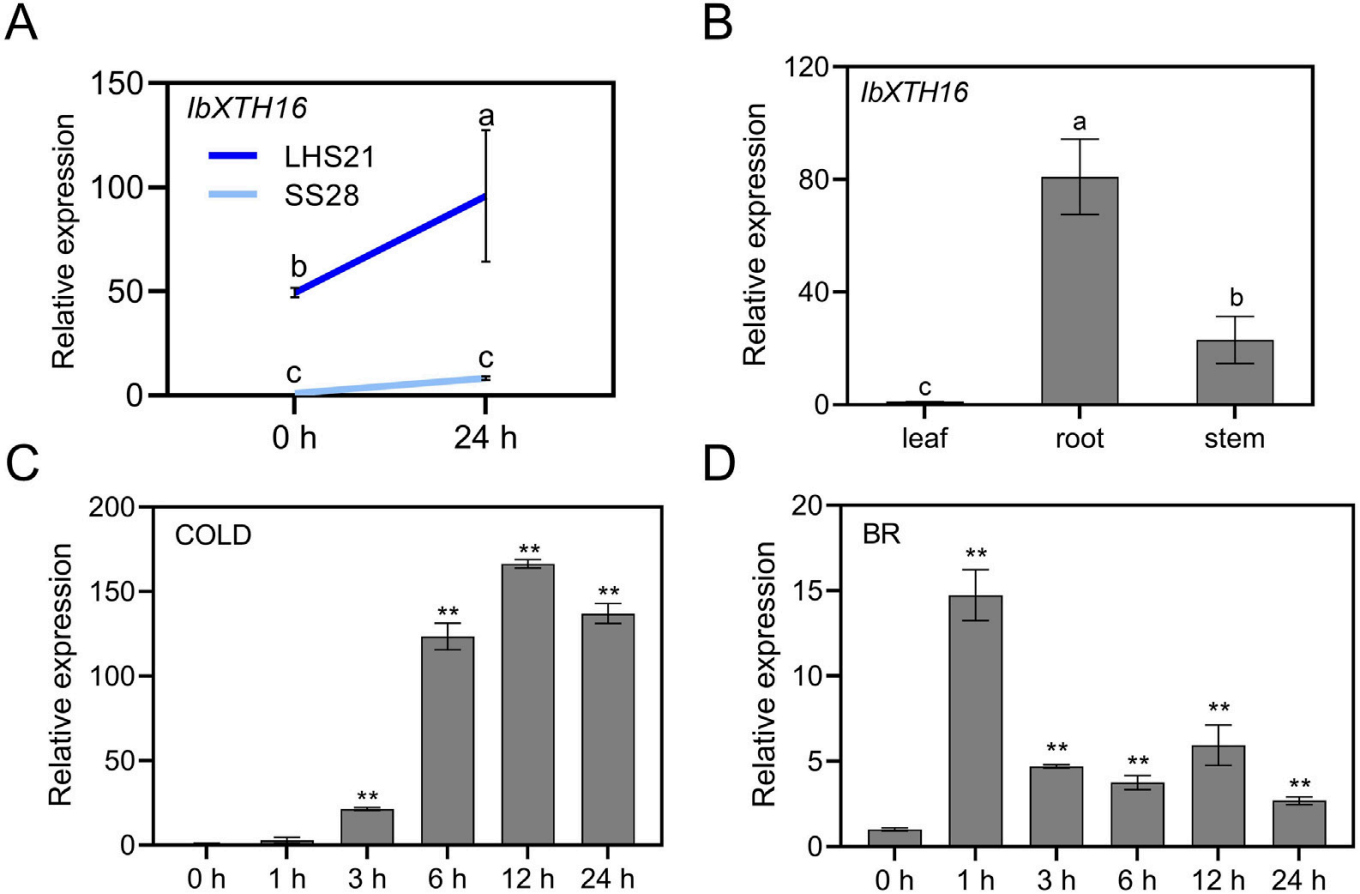
Expression analyses of *IbXTH16* in sweetpotato. **(A)** Expression of *IbXTH16* in cold-tolerant variety LHS21 and cold-susceptible variety SS28 under 4°C. **(B)** Expression of *IbXTH16* in 4-week-old *in vitro*-grown LHS21. **(C)** Expression of *IbXTH16* in cold-tolerant variety LHS21 after different time points (h) under 4°C. **(D)** Expression of *IbXTH16* in cold-tolerant variety LHS21 after different time points (h) in response to 100 mM BR. Different lowercase letters indicate differences at *P* < 0.05 based on one-way ANOVA followed by *post-hoc* Tukey’s test. ** indicates a significant difference at *P* < 0.01 according to Student’s *t*-test.

To study the potential role of *IbXTH16* in cold resistance of sweetpotato, the expression level of *IbXTH16* was analyzed. RT-qPCR assay showed that the expression level of *IbXTH16* in LHS21 was much higher than that in SS28 (Figure 2A). Tissue-specific expression assay revealed that the expression level of *IbXTH16* was relatively high in the roots of *in vitro*-grown LHS21 plants (Figure 2B). The expression of *IbXTH16* was significantly induced by 4°C and 100 mM BR (Figures 2C,D).

### 3.3 Overexpression of *IbXTH16* enhances cold tolerance in sweetpotato

**FIGURE 3:**
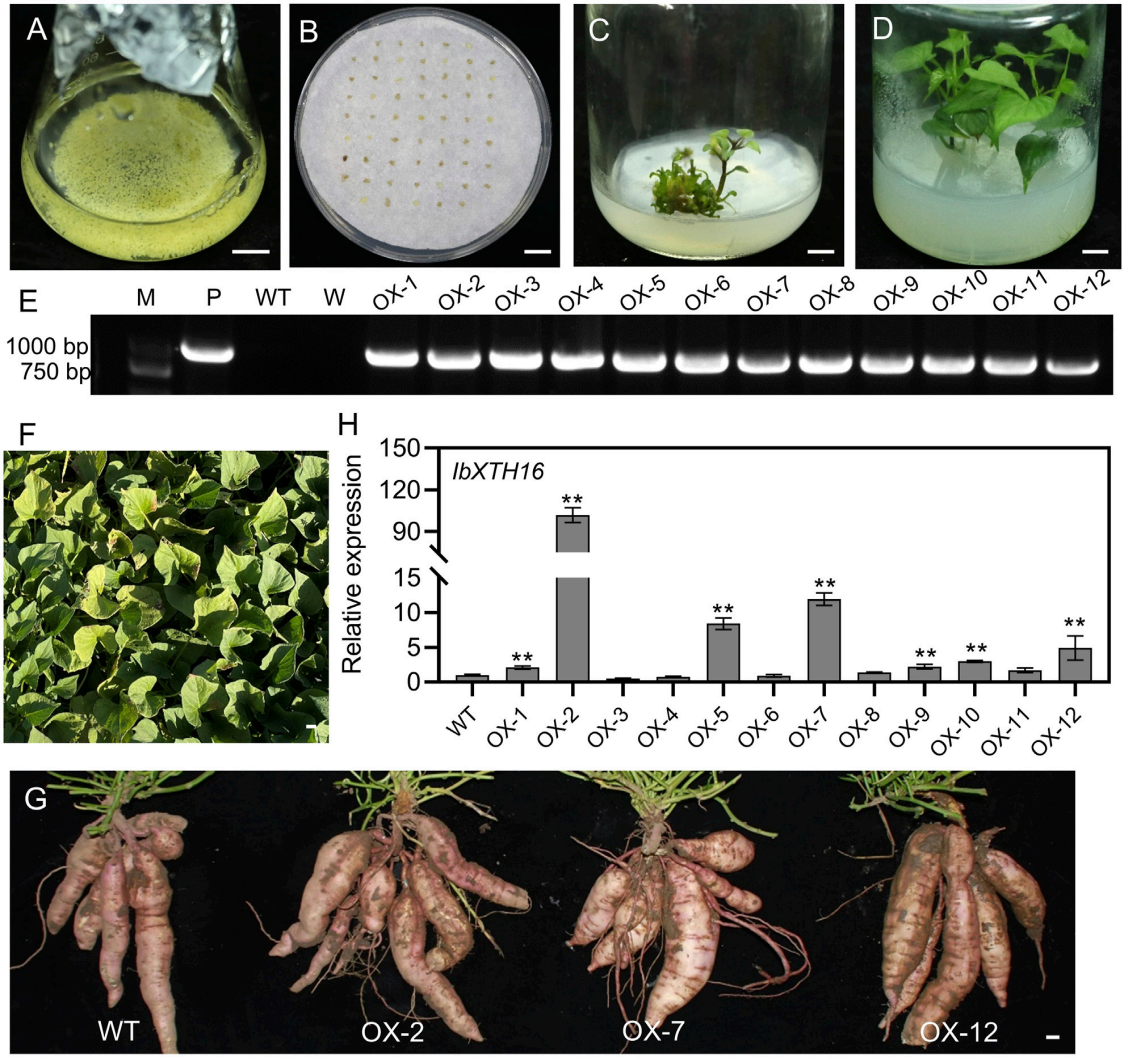
Production of the *IbXTH16*-overexpressing sweetpotato plants. **(A)** Lizixiang embryonic suspension cultures. **(B)** Screening of hygromycin-resistant embryogenic calli. **(C)** Regeneration of the *IbXTH16*-overexpressing plantlets. **(D)** Whole *IbXTH16*-overexpressing plants. **(E)** PCR identification of the *IbXTH16*-overexpressing plants. Lane M, DNA marker; Lane P, plasmid pCAMBIA1300-*IbXTH16* (positive control); Lane WT, Lizixiang (negative control); Lane W, water (negative control); OX-1-OX12, *IbXTH16*-overexpressing plants. **(F)** *IbXTH16*-overexpressing plants grown in a field. **(G)** Storage roots from WT and *IbXTH16*-overexpressing plants. **(H)** Expression analysis of *IbXTH16* in the overexpression plants by RT-qPCR. ** indicates a significant difference at *P* < 0.01 according to Student’s *t*-test. Bars = 1 cm.

**FIGURE 4:**
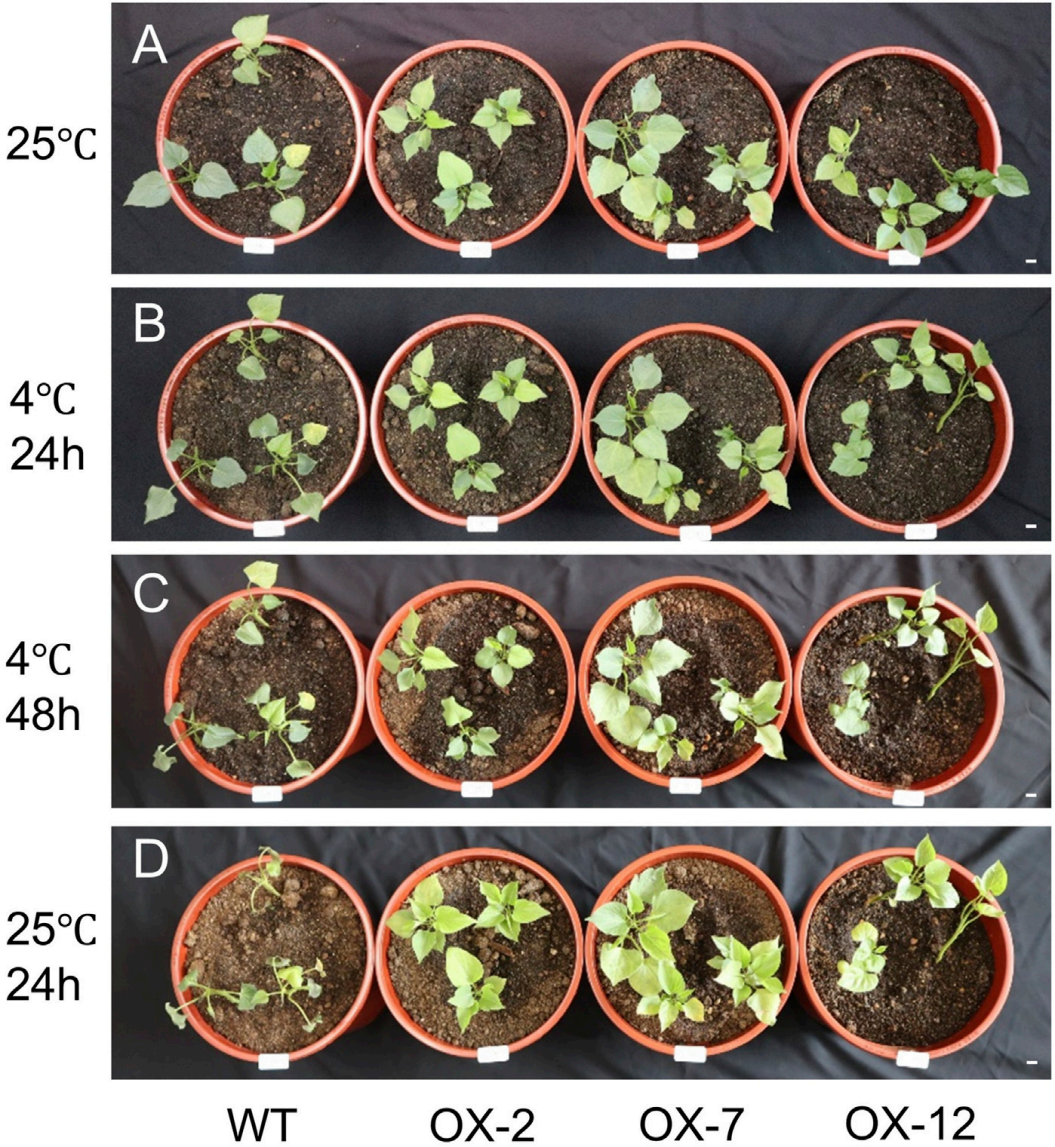
4°C treatment assays of the WT and *IbXTH16*-overexpressing sweetpotato plants. **(A)** Phenotype before 4°C treatments. **(B)** Phenotype after treatment at 4°C for 24 h. **(C)** Phenotype after treatment at 4°C for 48 h. **(D)** Phenotype after recovery at 4°C for 24 h. Bars = 1 cm.

To investigate whether *IbXTH16* contributes to cold tolerance in sweetpotato, this gene was transferred into sweetpotato variety Lizixiang via *A*. *tumefaciens*-mediated method, and 12 *IbXTH16*-overexpressing lines (OX-1 to OX-12) were generated (Figures 3A–F). There was no significant difference in the phenotype of sweetpotato storage roots between overexpression lines and WT (Figure 3G). *IbXTH16* exhibited increased expression level in the overexpression lines compared with the WT (Figure 3H). Three overexpression lines (OX-2, OX-7, and OX-12) with higher expression levels of *IbXTH16* were selected for further study. Furthermore, the overexpression and WT plants were treatment at 4°C and restored at 25°C. The degree of wilting in the overexpression lines at 24 h and 48 h under cold stress was lower compared to that of WT (Figures 4A–C). Additionally, the overexpression lines recovered more rapidly than WT when returned to 25°C (Figure 4D). These results indicated that *IbXTH16* functions as a positive regulator of cold tolerance in sweetpotato.

### 3.4 *IbXTH16* alters the contents of components and expression of genes related to stress response

**FIGURE 5:**
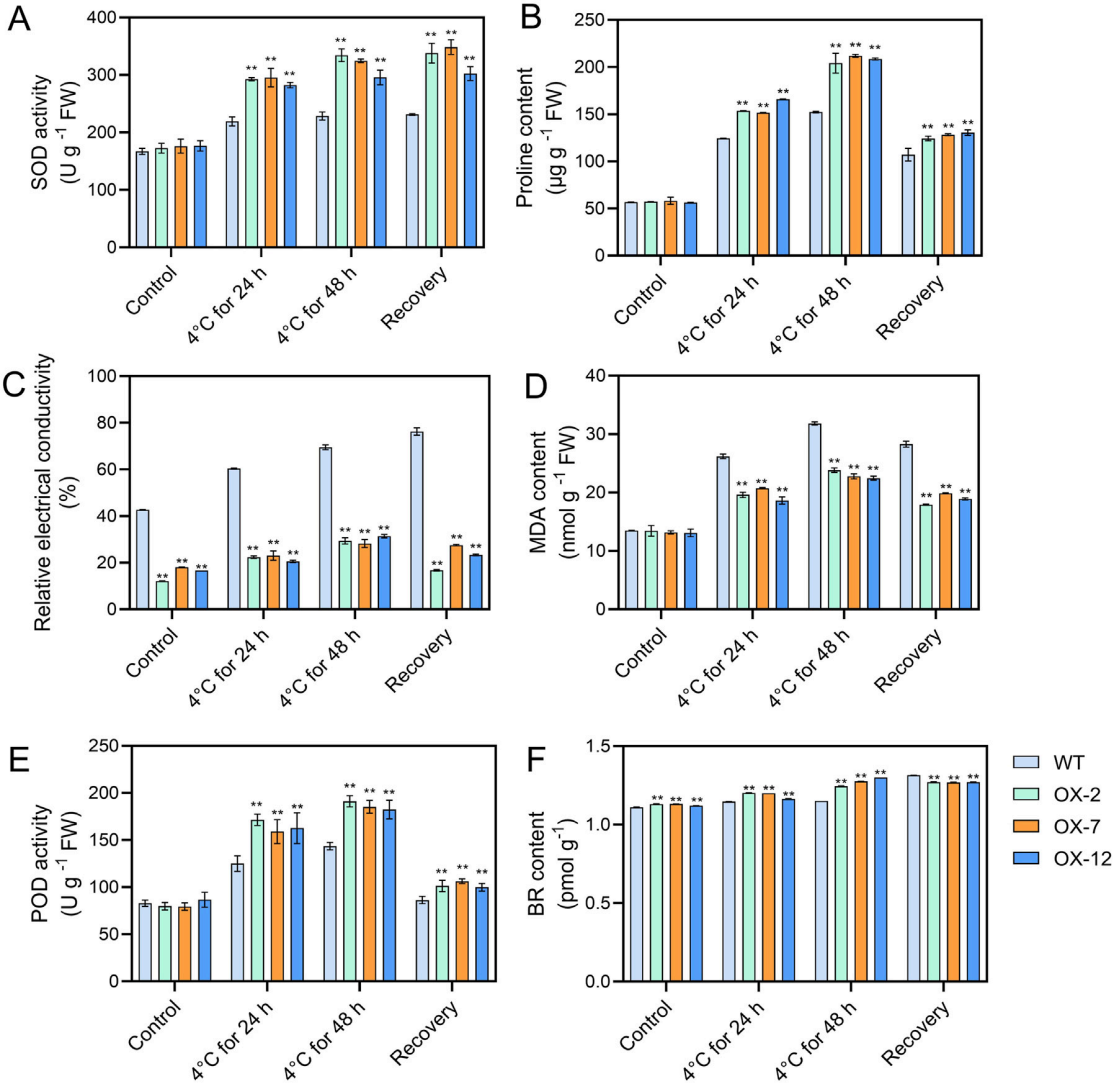
Analyses of components in the WT and *IbXTH16*-overexpressing sweetpotato plants. **(A)** SOD activity. **(B)** Proline content. **(C)** Relative electrical conductivity. **(D)** MDA content. **(E)** POD activity. **(F)** BR content. ** indicates a significant difference at *P* < 0.01 according to Student’s *t*-test.

**FIGURE 6:**
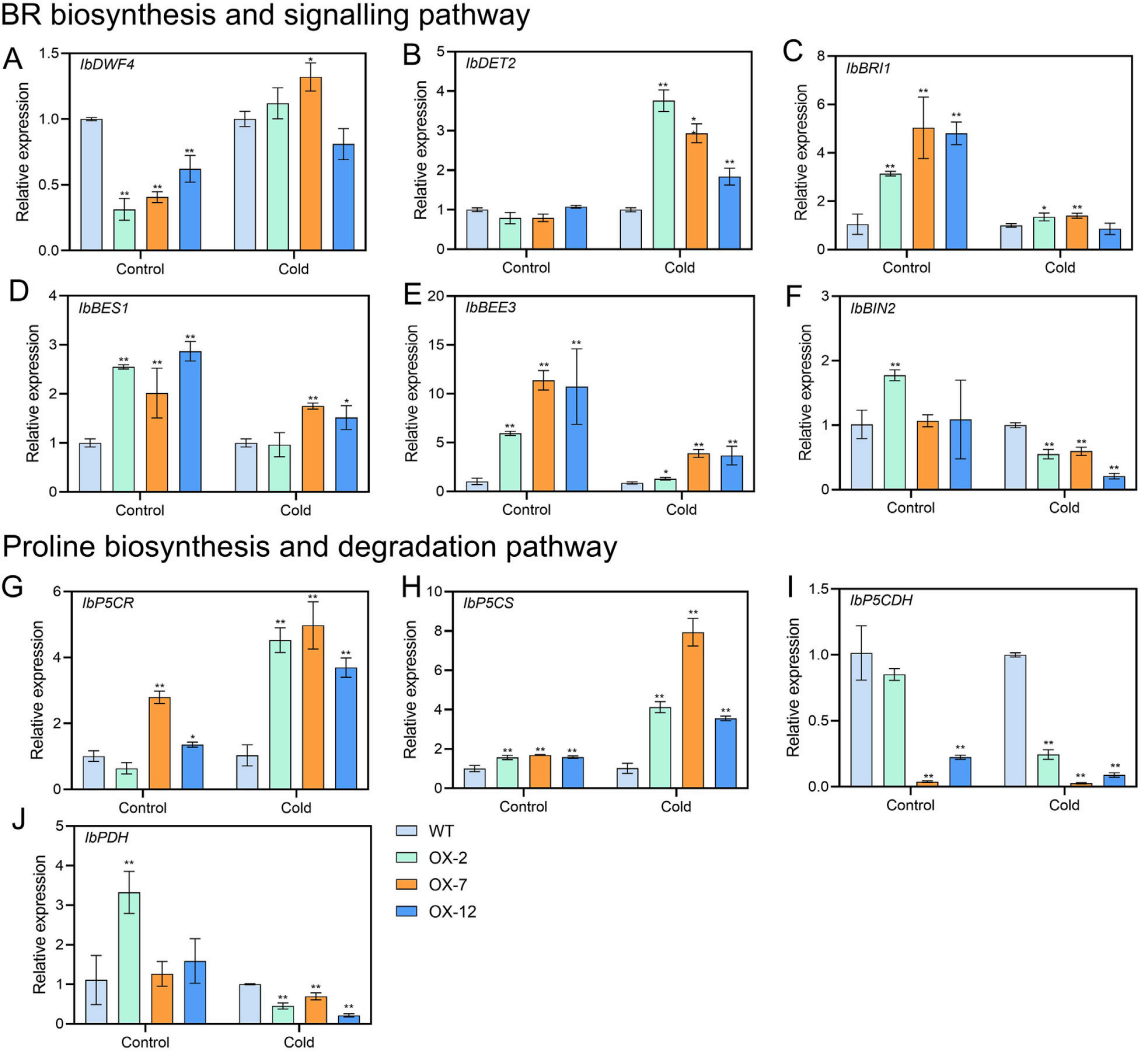
Expression analyses of BR and proline-related genes in the WT and *IbXTH16*-overexpressing sweetpotato plants under 4°C. **(A)** *IbDWF4*. **(B)** *IbDET2*. **(C)** *IbBRI1*. **(D)** *IbBES1*. **(E)** *IbBEE3*. **(F)** *IbBIN2*. **(G)** *IbP5CR*. **(H)** *IbP5CS*. **(I)** *IbP5CDH*. **(J)** *IbPDH*. * and ** indicate significant differences at *P* < 0.05 and *P* < 0.01 according to Student’s *t*-test.

To further explore how *IbXTH16* mediate the cold tolerance in sweetpotato, the contents of stress response-related components were measured. Under 4°C treatments for 24 h and 48 h, higher SOD and POD activities, higher proline and BR contents, and lower relative electrical conductivity and MDA content were found in the overexpression lines relative to WT (Figures 5A–F). In BR biosynthesis and signalling pathway, key enzyme genes *IbDWF4* and *IbDET2* and positive regulatory factors *IbBRI1*, *IbBES1*, and *IbBEE3* in the transgenic plants were upregulated under 4°C treatment (Figures 6A–E), while negative regulatory factor *IbBIN2* was downregulated (Figure 6F). In proline biosynthesis and signalling pathway, key enzyme genes *IbP5CR* and *IbP5CS* in the transgenic plants were upregulated under 4°C treatment (Figures 6G,H), while degradation pathway *IbP5CDH* and *IbPDH* were downregulated (Figures 6I,J). These results indicated that *IbXTH16* positively regulates cold tolerance of sweetpotato by activating the BR and proline pathways.

## 4 Discussion

### 4.1 *IbXTH16* positively regulates cold tolerance of sweetpotato

Many crops are well-suited for growth in tropical or subtropical regions (Chinnusamy et al., 2007). However, the average minimum temperature of most land areas on Earth is <0°C (Rihan et al., 2017). Low temperatures adversely affect crop growth and development, limiting their geographical distribution (Pearce, 2001; Ding et al., 2019). Sweetpotato is an important crop for ensuring national food security, but it is vulnerable to yield reductions caused by low-temperature damage (Jin et al., 2017; Yu et al., 2022). Genetic engineering has emerged as an effective strategy for enhancing sweetpotato’s tolerance to cold stress (Jin et al., 2017; 2021; 2022; Yu et al., 2022). Nevertheless, the function of XTH in cold stress of sweetpotato remains to be further studied. In this study, a novel *IbXTH16* gene was cloned from the cold-tolerant variety LHS21 (Figure 1). The plant cell membrane serves as a crucial barrier for maintaining stable cellular metabolism and also plays a key role in sensing low temperatures (Zhang et al., 2019). After plants are exposed to low temperatures, the permeability and fluidity of their cell membranes decline, activating cold-response genes (Muratan, 1997). The localization of IbXTH16 in the cell membrane and the induction of *IbXTH16* by cold suggest that IbXTH16 might serve as a signalling molecule in cold tolerance of sweetpotato (Figures 1D, 2C). The expression of *IbXTH16* was induced by the BR (Figure 2D), and its overexpression enhanced cold tolerance in sweetpotato (Figure 4). Therefore, *IbXTH16* is believed to be involved in the cold tolerance of sweetpotato.

### 4.2 *IbXTH16* activates the biosynthesis of SOD and POD

Under low-temperature stress, plants accumulate excessive reactive oxygen species (ROS), which can be detrimental to plant cells (Guo et al., 2022; Mittler et al., 2022). The ROS scavenging system can detoxify ROS by enhancing the activity of ROS-scavenging enzymes, such as SOD and POD, preventing oxidative damage to plant cells (Gill and Tuteja, 2010; Bose et al., 2014; Choudhury et al., 2017). In *Zoysia japonica*, overexpression of *ZjICE1* conferred cold tolerance in transgenic plants by increasing SOD, POD, and proline contents, as well as decreasing MDA content (Zuo et al., 2019). In *Betula platyphylla*, overexpression of *BpERF13* improved the cold tolerance of transgenic plants by binding to cis-elements of SOD and POD and increasing SOD and POD contents (Lv et al., 2020). In this study, the SOD and POD contents were significantly increased in the transgenic plants under 4°C (Figures 5A,E). It is suggested that overexpression of *IbXTH16* enhances cold tolerance by activating the biosynthesis of SOD and POD in transgenic sweetpotato. Previous reports have indicated that the plant hormone ABA is also involved in the cold tolerance of plants (Huang et al., 2017;Ma et al., 2018; Li et al., 2021). The *IbXTH16* promoter region contained an ABA-response element ABRE (Figure 1C). However, whether the *XTH16* gene regulates cold tolerance in sweetpotato via the ABA pathway requires further investigation.

### 4.3 *IbXTH16* positively regulates BR signalling pathway and proline accumulation

**FIGURE 7:**
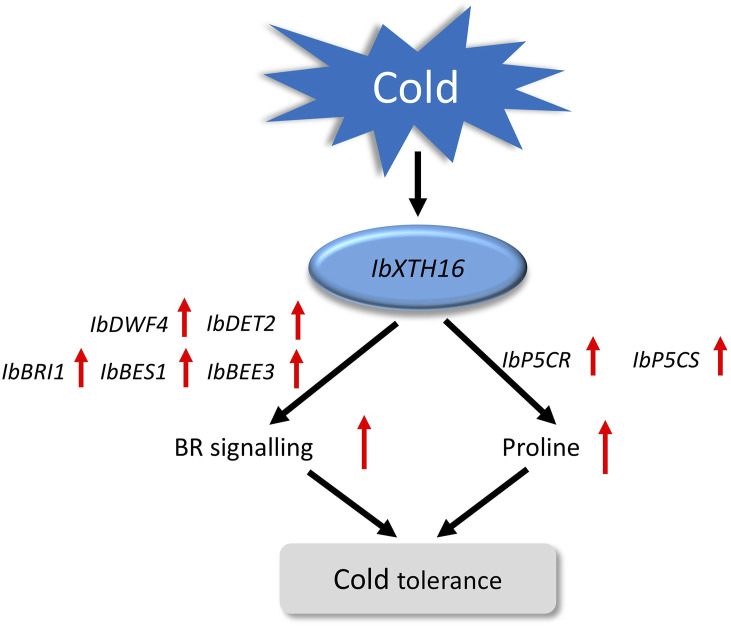
Proposed regulatory model of *IbXTH16* in the transgenic sweetpotato plants response to cold stress.

BR signalling not only participates in plant growth and development, but also has been reported in plant resistance to low temperature (Kinoshita et al., 2005; Vriet et al., 2012). In the initial phase when plants are exposed to cold stress, the activity of BIN2 kinase is suppressed, while OST1 kinase is activated, this synergistic regulation stabilizes ICE1, thereby enhancing the expression of *CBF* and improving the cold tolerance of plants (Barrero-Gil and Salinas, 2018; Ye et al., 2019). In *Arabidopsis*, compared with the WT, the cold resistance of overexpressing *BRI1* plants was enhanced, while that of mutant *BRI1* plants was decreased (Eremina et al., 2016). In this study, the transgenic plants showed a significant increase in BR content, which might be due to the overexpression of *IbXTH16* increasing the BR biosynthesis of transgenic plants, thus conferring cold tolerance (Figure 5F). Interestingly, the BR content in the transgenic plants was significantly decreased after restoring the room temperature (Figure 5F). The BR biosynthetic pathway involves the participation of a series of genes (Zhao and Li, 2012). In this study, the expression levels of BR biosynthesis and signalling pathway-related positive regulatory factors were significantly upregulated, while negative regulatory factor was significantly downregulated (Figures 6A–F). More proline accumulation can protect plants from low-temperature stress and ROS damage (Ghosh et al., 2022; Kidokoro et al., 2022). In this study, the proline biosynthesis-related genes were significantly upregulated, while degradation pathway-related genes were significantly downregulated (Figures 6G–J). Collectively, these findings suggest that *IbXTH16* positively regulates cold tolerance of sweetpotato by activiting BR signalling pathway and proline accumulation (Figure 7).

## 5 Conclusion

Overexpression of the cloned *IbXTH16* gene increased cold tolerance of sweetpotato by activating the BR and proline pathways. This study for the first time sheds light on the important role of *IbXTH16* in cold tolerance. *IbXTH16* has the potential to increase cold tolerance in sweetpotato and other plants.

## Data Availability

The original contributions presented in the study are included in the article/Supplementary Material, further inquiries can be directed to the corresponding author.
