# Supplementary material for: A xyloglucan endotransglucosylase/hydrolase gene, IbXTH16, increases cold tolerance in transgenic sweetpotato
Source: Front Genet. 2025 Jun 18;16:1629260. doi: 10.3389/fgene.2025.1629260 (PMC12213752; doi:10.3389/fgene.2025.1629260)
Supplement: Supplementary file 1 [file Table1.docx]

# Supplementary Table S1 – Primers used in this study.

| **Primer name** | **Primer sequence (5′–3′)** |
| --- | --- |
| *IbXTH16*-CDS-F | ATGGCATCTCAACTTTCCTTATT |
| *IbXTH16*-CDS-R | TCAGAATCTTGAGTTCTTGCACTCT |
| *IbXTH16pro*-F | GGCGGCGGACAGAATAA |
| *IbXTH16pro*-R | CATTTGATATATTTCTGAAGATCAG |
| pCAMBIA1300- *IbXTH16*-*GFP*-F | ACGGGGGACGAGCTCGGTACCATGGCATCTCAACTTTCCTTATT |
| pCAMBIA1300- *IbXTH16*-*GFP*-R | CATGTCGACTCTAGAGGATCCGAATCTTGAGTTCTTGCACTCT |
| *IbActin*-qF | AGCAGCATGAAGATTAAGGTTGTAGCACT |
| *IbActin*-qR | GGAAAATTAGAAGCACTTCCTGTGAAC |
| *IbXTH16*-qF | CTGCAACTTCTTATGGCGGC |
| *IbXTH16*-qR | CCTGCATCACTTTACCCCGT |
| *pCAMBIA1300-IbXTH16*-F | ACGGGGGACGAGCTCGGTACCATGGCATCTCAACTTTCCTTATT |
| *pCAMBIA1300-IbXTH16*-R | CATGTCGACTCTAGAGGATCCGAATCTTGAGTTCTTGCACTCT |
| 35S-F | GCACAATCCCACTATC |
| *IbXTH16*-R | TCAGAATCTTGAGTTCTTGCACTCT |
| *IbDWF4*-qF | ATGGCTGGAGATCAAAGGTGG |
| *IbDWF4*-qR | AAGTAATTCAGGTCTTGGTCACATG |
| *IbDET2*-qF | ATGATGTCTATCCGAGAAGTATTGG |
| *IbDET2*-qR | GAGTTCCTGCCAAAAGGGAAT |
| *IbBRI1*-Qf | TAGCGTGGTTCCAACATCCC |
| *IbBRI1*-qR | TGCCTTCATCGCAACCTTCT |
| *IbBES1*-qF | AAGCTGGTTGGGTTGTCGAT |
| *IbBES1*-qR | GGACTGGGAAAGCACGATGA |
| *IbBEE3-*qF | AGCCTGCAGATTACACCACC |
| *IbBEE3*-qR | TGTGACTATCAGTGGCCTGC |
| *IbBIN2*-qF | TGCCACTAGGACCTCAGCC |
| *IbBIN2*-qR | TTCTGCCATGTAACTAATTGTCCTT |
| *IbP5CR*-qF | ATAGAGGCATTGGCTGATGG |
| *IbP5CR* -qR | GGTAGTCCCACCTGGTGATG |
| *IbP5CS*-qF | GCCTGATGCACTTGTTCAGA |
| *IbP5CS*-qR | TTGAGCAATTCAGGGACCTC |
| *IbP5CDH*-qF | TCAGTTACAAGCGTTCACACAGT |
| *IbP5CDH*-qR | TGCAACTTTAATAAATGACTCCCCA |
| *IbPDH*-qF | GGGGGTATGAGTGGAAAAGTGTA |
| *IbPDH*-qR | ATCAGCCTCCTTAGCACATCCTC |
