# Supplementary material for: A xyloglucan endotransglucosylase/hydrolase gene, IbXTH16, increases cold tolerance in transgenic sweetpotato
Source: Front Genet. 2025 Jun 18;16:1629260. doi: 10.3389/fgene.2025.1629260 (PMC12213752; doi:10.3389/fgene.2025.1629260)
Supplement: Supplementary file 2 [file Table2.docx]

**Supplementary Table S2–The sequence of *IbXTH16***

**ATG**GCATCTCAACTTTCCTTATTCCTGCAACTTCTTATGGCGGCTTGTTTGGTGGCAGCCGCCATGGCTGCAAATTTCAACCAAGACGTTCAGATGTATTTTGGCAATGGACGGGGTAAAGTGATGCAGGGCGGCACCATGGCAGCTCTTACTCTCGACAGAGAATCAGGTTCCGGCTTCCAATCCACCAACGAATATCTTTTCGGCAGATTTGATATGCAGGTCAAGCTTATCTCTGATAACTCTGCTGGAACCGTTACTACTTTCTACTTATCTTCTCTAGGAGACAGACATGACGAAATTGACTTCGAATTCCTGGGCAATGTCTCCGGCCAGCCTTACACAATCCACACCAATGTATATTCTCAAGGGAAAGGAGGCAGGGAGCAGCAATTCCATCTCTGGTTCGACCCCACCACCGCCTACCACACTTACTCCATTGTTTGGAACTCTCAGCGCATCATCTTCCTCGTCGACAACATTCCCATCAGAGTTTACCGGAACCACGAGAGCATGGGGGTCCCTTTCCCCAAGAATCAGCCCATGAGAGTCTACTGCAGCTTATGGAACGCAGACGACTGGGCTACGCAGGGGGGCCTTGTCAAGACTGATTGGACTAAGGCACCCTTCACTGTTTACTACCGGAACTTCAATATTGATGCCTGTGTCGTCTCCGGCGGTCGGTCGTCTTGCGATTCCAAGTCGTCCGCCGATCCGGTGAACAACAAACAGGCGTGGCAGACGCAGGATGTTGATGCGCGTGGGAGGAATAGGTTGAGATGGGTGCAAAGCAAACATATGGTTTACAATTACTGTGCTGATTCCAAGAGGTTTCCTGGAGGTACTTTTCCTGCAGAGTGCAAGAACTCAAGATTC**TGA**
